# Transfer cells

**DOI:** 10.3389/fpls.2014.00672

**Published:** 2014-11-26

**Authors:** David W. McCurdy, Gregorio Hueros

**Affiliations:** ^1^Centre for Plant Science, School of Environmental and Life Sciences, The University of NewcastleNewcastle, NSW, Australia; ^2^Departamento Biomedicina y Biotecnología (Genética), Universidad de AlcaláMadrid, Spain

**Keywords:** transfer cells, cell wall ingrowths, basal endosperm transfer layer, syncytia, phloem parenchyma transfer cells, giant cells

Advances in plant science research will lie at the center of efforts to address global food security as demand for agricultural production is predicted to more than double by 2050. A key determinant of plant productivity and thus crop yield is efficient nutrient transport between sites of net nutrient synthesis and acquisition to sites of net utilization. Transfer cells (TCs) play key roles in optimizing such nutrient transport processes in plants, and thus research on these anatomically specialized cell types has the potential to contribute new approaches for improving plant performance. The collection of reviews and research articles in this Research Topic on Transfer Cells provides a valuable contribution to advancing our understanding of these important cell types in plant biology.

TCs *trans*-differentiate from existing cell types by developing extensive wall ingrowths. The resulting increase in plasma membrane surface area enables increased densities of membrane transporters to optimize nutrient transport across apoplasmic/symplasmic boundaries at sites where TCs form. Key challenges in TC biology include understanding the signals and genetic pathways required for initiating and building the unique wall ingrowths that define TC identity. This knowledge in turn will provide a basis to investigate physiological and genetic factors explaining the variability observed in TC differentiation, even among equivalent anatomical situations in highly related species.

Amongst many other scenarios, TCs are involved in delivery of nutrients between generations and, as evidenced by the number of contributions in this Research Topic, considerable focus is being directed toward investigating endosperm TCs in seeds of cereals such as maize and barley. Thiel (2014) provides a comprehensive review of the development of these cells in barley, including transcript profiling studies which have revealed a likely role for two-component signaling pathways in endosperm TC differentiation, in addition to identifying a developmental switch across grain filling from active to passive modes of nutrient uptake as revealed by expression profiles of membrane transporter genes. Lopato et al. (2014) review the field of endosperm TC-specific genes and their encoded proteins, emphasizing the potential for using promoters of such genes for biotechnological applications. Identifying the repertoire of genes showing TC-specific expression provides important molecular insight into these cell types, and two research articles contribute to this goal. Royo et al. (2014) describe the non-overlapping expression of *BETL9* and *BETL9like*, two lipid transfer genes similar to *END-1* expressed specifically in barley endosperm TCs. While the functions of these genes remain unknown, *BETL9* is expressed exclusively in the basal endosperm transfer layer (BETL), but protein product accumulates in maternal tissue adjacent to this layer. In contrast, *BETL9like* is expressed specifically in the endosperm aleurone layer. The expression domains of these genes completely surround the filial tissues, suggesting a protective role at the maternal-filial interface. The *miniature*1 (*mn*1) mutant in maize is defective in a BETL-specific cell wall invertase (INCW2), resulting in reduced hexose levels and wall ingrowth development in this tissue. Silva-Sanchez et al. (2014) report a comparative glycoproteome analysis of developing endosperm in *mn*1 seed compared to the wild type *Mn*1. Most of the identified proteins showing decreased glycosylation in *mn*1 were involved in post-translational modification, protein turnover, chaperone function, carbohydrate metabolism and cell wall biosynthesis, suggesting links to endoplasmic reticulum stress and the unfolded protein response in the mutant due to compromised glycosylation levels. Muñiz et al. (2014) describe establishment of a PCR-based forward genetic screen using expression domain-specific markers to identify new mutant lines showing altered TC development. Application of this forward genetics approach using genes known to be tissue or developmental-stage specific offers new tools to extend the use of maize as a model for TC research. Finally, Rocha et al. (2014) report the unexpected finding that both reticulate-like and flange wall ingrowths in maize BETL contain significant amounts of lignin, a result counter-intuitive with the presumed need for high rates of nutrient diffusion through the wall ingrowths themselves (see Gunning and Pate, 1974) and the general role of lignification in providing cell wall rigidity and strength. A reassessment of wall ingrowth structure and ontogeny is needed in light of this finding.

Upon infection of root tissue, cyst and root-knot nematodes induce vascular cells to form enlarged feeding cells, termed syncytia and giant cells, respectively. These cell types both form wall ingrowth structures reminiscent of those seen in TCs, and are thought to function similarly to facilitate enhanced rates of apoplasmic/symplasmic solute exchange required for feeding the invading nematode. Rodiuc et al. (2014) review the cellular modifications and transport functions of these nematode feeding sites in Arabidopsis roots, while Cabrera et al. (2014) review transcriptomic signatures of these cells in Arabidopsis with emphasis on auxin and ethylene signaling pathways. In contrast to endosperm TCs in cereals, genes involved in two-component signaling appear not to contribute significantly to the development of nematode feeding cells, nor are genes associated with early signaling involving reactive oxygen species.

Arabidopsis provides a powerful genetic system to study TC biology (Arun Chinnappa et al., 2013). Phloem parenchyma TCs of leaf minor veins form wall ingrowths which are involved in phloem loading (Haritatos et al., 2000) by facilitating apoplasmic unloading of sucrose via the recently discovered sucrose effluxers AtSWEET11 and 12 (Chen et al., 2012). The research paper by Maeda et al. (2014) analyzes the role of callose synthases in TC wall development in the tocopherol deficient mutant *vte2*. When grown at low temperature, *vte2* shows reduced photoassimilate export from leaves due to massive callose deposition specifically in phloem parenchyma TCs. Transcript profiling and knockout studies revealed the confounding result that while *GLUCAN SYNTHASE LIKE 4* (*GSL4*) and *GSL11* were induced specifically at low temperature in *vte2*, only disruption of *GSL5* substantially reduced this phloem parenchyma TC-specific production of callose, but had no effect on the low temperature photoassimilate export phenotype of *vte2*. Adams et al. (2014) report a significant correlation between photosynthetic capacity and wall ingrowth development in minor-vein phloem parenchyma (Arabidopsis and *Senecio*) and companion TCs (pea and *Senecio*), consistent with the role of these TCs in supporting photoassimilate export from leaves. In contrast, responses to stress such as application of methyl jasmonate caused increased wall ingrowth deposition in phloem parenchyma TCs alone, indicating cell specific responses of TCs to different signals and possibly different roles for these TCs (sugar export vs physical defense against pathogen infection).

Collectively, the insights into TCs provided in the reviews and research articles assembled for this Research Topic establish a valuable platform for continued investigation of these fascinating cell types in plants. Further research on TCs may unlock key possibilities for improving crop yield and thus contribute to the rapidly approaching challenge of addressing global food security.

## Conflict of interest statement

The authors declare that the research was conducted in the absence of any commercial or financial relationships that could be construed as a potential conflict of interest.
